# Pulmonary hypertension in a patient with HHV-8–negative multicentric castleman disease and epstein-barr virus co-infection: a case report

**DOI:** 10.3389/fmed.2026.1951176

**Published:** 2026-10-14

**Authors:** Xi Yan, Lipin Xie, Hongrui Li, Jiao Tang, Maoyun Wang, Lan Wang

**Affiliations:** 1 Department of Respiratory and Critical Care Medicine, West China Hospital, Sichuan University, Chengdu, China; 2 Department of Hematology, West China Hospital, Sichuan University, Chengdu, China; 3 Department of Respiratory and Critical Care Medicine, The People's Hospital of Liuyang City, Liuyang, China

**Keywords:** case report, castleman disease, epstein–Barr virus, pulmonary hypertension, siltuximab

## Abstract

Pulmonary hypertension (PH) seems to be a rare but severe complication of Castleman disease (CD), especially the multicentric type (MCD). We report a case of recurrent fever, anemia and progressive dyspnea. She was finally diagnosed with pre-capillary PH in the setting of MCD and Epstein–Barr virus (EBV) co-infection after right heart catheterization and lymph node biopsy. After receiving siltuximab, an anti-interleukin-6 monoclonal antibody along with diuretic agent, the symptoms were relieved. However, the patient died 8 months after diagnosis due to a pulmonary hypertensive crisis secondary to clinically diagnosed bacterial pneumonia presenting with severe dyspnea, refractory hypoxemia, and acute right ventricular failure. This rare case improves recognition of this infrequent etiology of PH.

## Introduction

Pulmonary hypertension (PH) is a rare but potentially life-threatening disease which diminishes the patients' physical activity, increases the risks of heart failure and imposes significant socio-economic burdens (1, 2). Recognizing the etiology is crucial to efficient treatment; however, it remains a complex and intricate procedure. In recent years, some uncommon factors such as viral infections and multi-system diseases have been reported linked with PH (3, 4) thereby improving our understanding of this heterogeneous disease.

Castleman disease (CD) is a rare lymphoproliferative disorder characterized by lymphadenopathy and a variable systemic inflammatory response (5). It is subclassified into localised CD, which typically involves a single enlarged lymph node, and multicentric CD (MCD), which presents with generalized lymphadenopathy, hepatosplenomegaly, and constitutional symptoms such as fever, weight loss, sweats, and anemia (6). MCD can be further stratified by human herpesvirus 8 (HHV-8) status; HHV-8-negative MCD, also known as idiopathic MCD（iMCD), is less common and often associated with dysregulated cytokine production, particularly interleukin-6 (IL-6) (7).

To date, only a few cases have been reported the link between MCD and PH (8, 9). Most of the existing literature focuses on HHV-8-positive MCD, whereas PH in the setting of HHV-8-negative MCD with chronic Epstein–Barr virus (EBV) co-infection has never been described. The corresponding therapeutic approach also remains absent.

Here we report a rare case of pre-capillary PH in a 44-year-old female patient with HHV-8-negative, HIV-negative MCD and EBV co-infection. The patient presented with recurrent fever, lymphadenopathy, and progressive dyspnea within one year. After receiving the treatment with prednisone and siltuximab, an anti-interleukin-6 monoclonal antibody, the symptoms were partially relieved. This case describes the detailed clinical presentation, diagnostic challenges, potential pathogenic role of IL-6 and IgE, and therapeutic considerations, including the use of siltuximab and macitentan. By reporting this case, we aim to improve awareness of MCD as an underrecognized cause of PH and to share our experience in early recognition and management.

## Case description

A 44-year-old female patient complained of recurrent fever and lymphadenopathy for two months without special medical, family and psychosocial history. Laboratory tests showed hemoglobin 99 g/L and the Epstein–Barr virus (EBV) load 190 copies/mL. Ultrasound examination confirmed inguinal, cervical and axillary lymphadenopathy. Bone marrow smear and biopsy, as well as the inguinal lymph node biopsy excluded a hematological disorder; autoimmune disease screening was also negative. She was then diagnosed with EBV infection and treated with acyclovir tablets for 3 months with resolution of fever. During the following 9 months after discharge, the patient continued to have recurrent fever and lymphadenopathy despite a decrease in EBV level to less than 50 copies/mL (shown in Figure 1).

**Figure 1:**
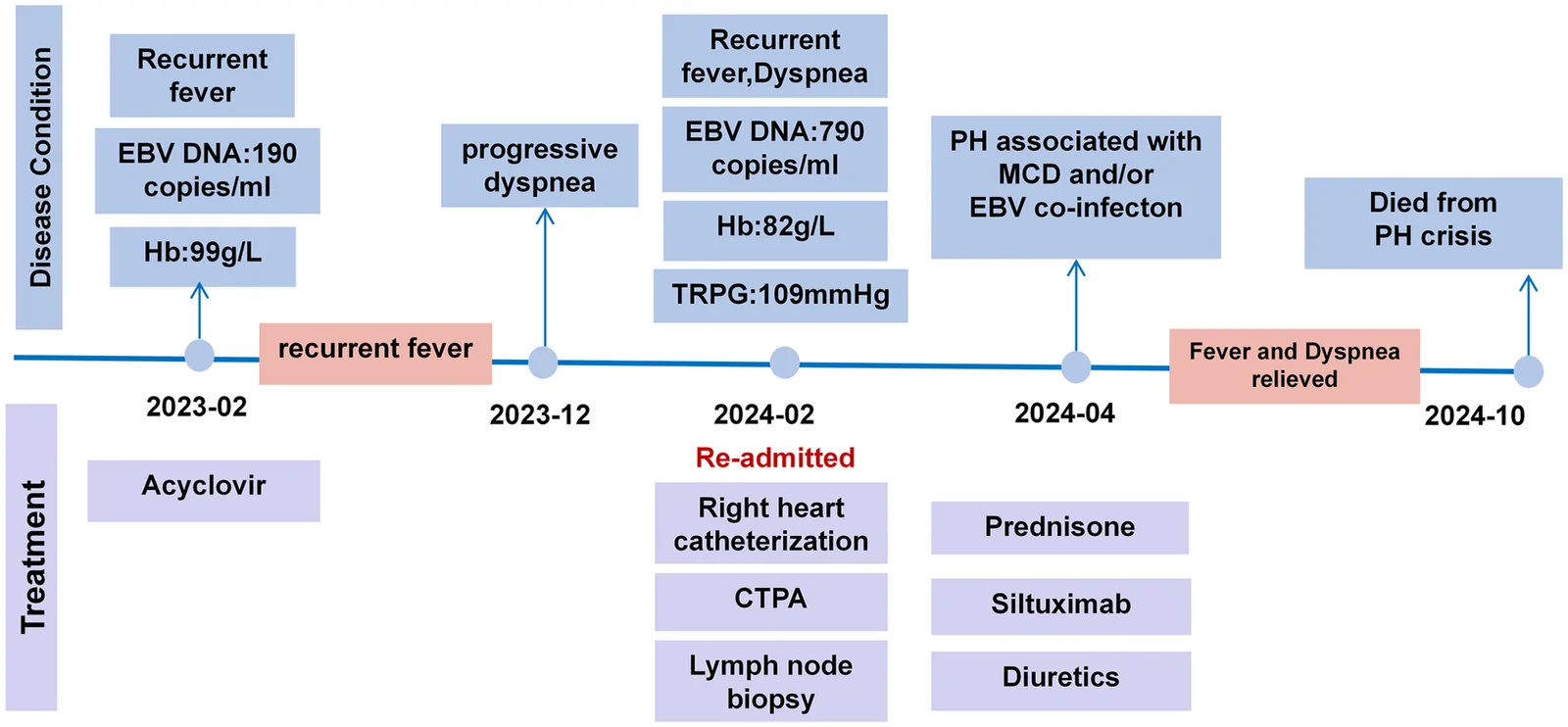
The timeline of the disease condition and treatment. EBV: Epstein–Barr virus; Hb: hemoglobin; TRPG: Tricuspid regurgitation pressure gradient; CTPA: Computed tomography pulmonary angiography; MCD: Multi-center Castleman disease.

Three months later, the patient developed progressive dyspnea on moderate exertion, and was readmitted. Physical examination: temperature 38.3 °C, respiratory rate 22 breaths/min, pulse oxygen saturation 95% on 3 L/min oxygen. The lymph nodes in the cervical, axillary, and inguinal areas and the enlarged spleen could be palpated. Coarse breathing sounds and obvious heart murmur near the tricuspid valve area could be heard. Routine laboratory findings: Hb 82 g/L and a white blood cell count 12.14  ×  10^9^/L (neutrophil 79.5%). serum IL-6 83.58 pg/mL, N-terminal prohormone of brain natriuretic peptide (NT-pro BNP) 6167 pg/mL, vascular endothelial growth factor (VEGF) 164.13pg/mL. The copies of EBV-DNA were 790 copies/mL and IgE was 11800 IU/mL. An arterial blood gas analysis demonstrated hypoxemia and the oxygenation index was 210. The echocardiogram revealed that right heart enlargement and tricuspid regurgitation (TR) pressure was 109 mmHg compared to normal echocardiogram ten months earlier (Figure 2). The result of right heart catheterization showed the pulmonary artery pressure (PAP) was 70/36 (47) mmHg, pulmonary artery wedge pressure (PAWP) was 11/1 (5) mmHg, cardiac output (CO) was 3.8 L/min, cardiac index (CI) was 2.7 L/min/m^2^ and pulmonary vascular resistance (PVR) was 11.05 Wood units (WU), which confirmed pre-capillary PH. Computed tomography pulmonary angiography (CTPA) illustrated a thickened pulmonary artery trunk without filling defects or pulmonary stenosis, excluding the group 4 PH caused by chronic thromboembolic pulmonary hypertension (CTEPH), Takayasu arteritis, or echinococcosis. PH due to left heart disease, lung disease/hypoxia, pulmonary veno-occlusive disease, and known causes of group 1 PH (congenital heart disease, connective tissue disease, parasites, heredity, toxins, drugs) was all ruled out. Given her persistent fever and lymphadenopathy with EBV infection, group 5 PH associated with hematological diseases was suspected. Her six-minute walk distance was 259 m, with a Borg dyspnea score of 6.

**Figure 2:**
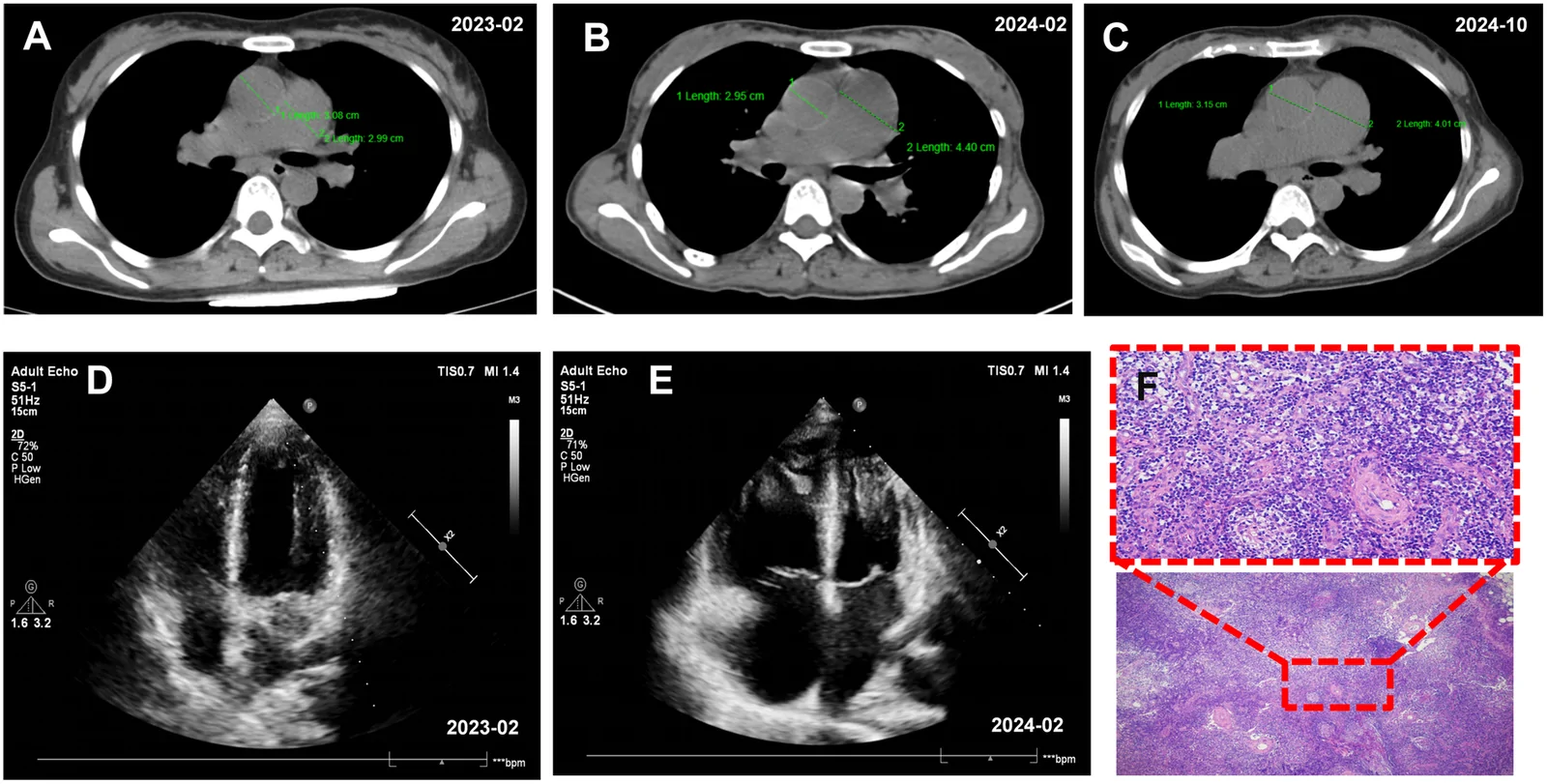
**(A–C)** CT images of the patient the first admitted (202302), before treatment (202402) and post-treatment (202410). **(D)** An echocardiogram when the patient was the first admitted (202302) showed the normalization of right atrial (RA) and ventricular cavities (RV). **(E)** An echocardiogram when the patient was the readmitted showed dilated right atrial and ventricular cavities (202402). **(F)** Lymph node biopsy and Lymph node with evidence of Castleman disease.

To establish the underlying diagnosis, an axillary lymph node biopsy was performed. Histological examination of the right cervical lymph node biopsy revealed a lymphoproliferative lesion. Morphological features included partial atrophy of germinal centers, small vessels penetrating the follicles, and focal hyalinization of the vascular walls. Immunohistochemistry demonstrated the following profile: CD20 (partially+), CD3 (partially+), CD23 (follicular dendritic cell network+), CD6 (partially +), CD10(-), CD138(partially+), and IgG4(scattered+). The Ki-67 (MIB-1) proliferation index was approximately 20%–30%. *In situ* hybridization for Epstein–Barr virus-encoded RNA (EBER) showed only scattered positive cells, and HHV-8 was negative (Figure 2F). Flow cytometry detected no abnormal T, B, or NK lymphocyte populations. Based on the characteristic features of atrophic germinal centers, hyalinized vascular walls (classic hyaline vascular morphology), and a partial plasma cell component (CD138+), the histopathological subtype was classified as hyaline vascular-predominant/mixed type. After multidisciplinary consultation, she was diagnosed with HHV-8(–), HIV (–) multicentric Castleman disease (MCD) and EBV infection. The PH was considered most likely secondary to MCD and/or possible EBV co-infection.

She then received prednisone 20 mg/day and intravenous siltuximab 11 mg/kg every three weeks which is the first-line target therapy recommended to treat iMCD according to the evidence-based consensus treatment guidelines and clinical trials (10, 11), plus oral diuretics furosemide 20 mg/day and spironolactone 20 mg/day for right heart dysfunction over three months. Fever resolved and dyspnea partially improved; six-minute walk distance increased. Hemoglobin recovered to 127 g/L; IL-6 decreased to 50.9 pg/mL, NT-pro BNP to 1639 pg/mL (shown in Table 1). However, echocardiographic parameters of right heart size and estimated pulmonary arterial pressure did not improve markedly. 10 mg of macitentan per day was added, leading to further symptomatic relief. The patient demonstrated good adherence to the prescribed regimen and no significant treatment-related adverse events were observed during the following three months. However, the patient died 6 months after diagnosis due to a pulmonary hypertensive crisis secondary to lung infection presenting with severe dyspnea, refractory hypoxemia, and right heart failure. Despite intensive supportive therapy, the patient ultimately died of acute right heart failure.

**Table 1:** Summary of clinical, laboratory, and echocardiographic parameters.

Parameters	Stage 1 (202306)	Stage 2 (202402)	Stage 3 (202407)	Reference
Hb	99	82	127	115-150 g/L
CRP	29	75	8.8	0-6 mg/L
PCT	0.06	0.17	0.06	<0.045 ng/mL
IL-6	44.9	83.6	50.9	-
EBV	190	797	<50	copies/mL
NT-pro BNP	/	6167	1639	<226 ng/L
NYHA functional class	Ⅰ	Ⅳ	Ⅱ	-
6MWD(m)	/	259	420	-
Echocardiogram				
LVD	40	40	36	-
LAD	24	26	23	-
RVD	22	29	33	-
RAD	23	42	51	-
EF (%)	66%	67%	76%	-
TRV(m/s)	/	5.2	4.4	-
TRG (mmHg)	/	109	78	-

Stage 1: the patient had fever and anemia symptoms, but did not feel dyspnea. Stage 2: the patient felt dyspnea but did not receive the treatment. Stage 3: the patient received the targeted therapy. Hb: hemoglobin; PCT, procalcitonin; CRP, C-reactive protein; NT-pro BNP, brain-natriuretic peptide, EBV, epstein–barr virus; NYHA functional class: New York Heart Association (NYHA) functional class; 6MWD, six minutes' walk distance; LAD, left atrial diameter; RAD, right atrial diameter; LVD, left ventricle diameter; RVD, right ventricle diameter; EF, ejection fraction; TRV, tricuspid regurgitation velocity; TRPG, tricuspid regurgitation pressure gradient.

## Discussion

MCD is a rare lymphoproliferative disorder characterized by systemic lymphadenopathy, hepatosplenomegaly and constitutional symptoms such as fever, anemia and weight loss (5). PH seems to be an infrequently recognized but fatal complication of MCD with only a few reported cases (8, 9, 12, 13). We present a rapidly progressive case of severe pre-capillary PH in a patient with HHV-8 (–), HIV (–) MCD but EBV co-infection. After receiving diuretics, macitentan and siltuximab (anti-IL-6 antibody), the symptoms such as recurrent fever, anemia and dyspnea were relieved. However, the patient suffered from pulmonary crisis secondary to bacterial pneumonia.

The potential mechanisms linking MCD and PH may include: overproduction of IL-6, a key pathogenic factor in MCD, promotes pulmonary vascular remodeling in animal models and humans through the JAK/STAT3 pathway (14–16). Treatment with siltuximab (anti-IL-6 antibody) reduced IL-6 levels and partially relieved symptoms, supporting the causal role. Notably, the abnormal echocardiographic parameters failed to improve, suggesting that once structural remodeling which was driven by chronic IL-6/JAK/STAT3 activation, only IL-6 blockade is insufficient to reverse PH. Additionally, polyclonal hypergammaglobulinemia (elevated IgE as seen in our patient) may also contribute to PH via hyperviscosity and mast-cell activation, which further releases IL-6 and IL-13 amplifying inflammation and fibroblast activation (17, 18). This elevated IgE, not associated with hyper-IgE syndrome, allergic bronchopulmonary aspergillosis and parasitic infections evidenced by clinical examination may explain the persistent PH despite partial IL-6 suppression. In conclusion, abnormal immune cell function and cytokine storm of MCD influence the pathophysiology of PH.

Among previously reported PH cases associated with CD, only one fatal case was identified with HHV-8 positive in the lung tissue and she presented sign of right heart failure including non-productive cough, dyspnea and lower extremity oedema (8). It is suspected that HHV-8 may stimulate VEGF via a viral IL-6 homolog, driving angioproliferation and progression of PH (19). Our patient was HHV-8 negative but still died, suggesting that cytokine dysregulation and possibly EBV co-infection may lead to poor outcomes as reported in previous cases (16). Although EBV DNA levels were low and fluctuating and EBER was only scattered, the vascular endothelial damage and inflammation induced by EBV may still be involved in progression of PH (17). To contextualize our findings, we compared our case with 16 previously reported cases of PH associated with EBV and/or Castleman disease (Supplementary Table 1). Among these, most patients achieved both CD/EBV resolution and PH improvement when the underlying disease was controlled, particularly in those receiving intensive immune-chemotherapy or hematopoietic stem cell transplantation. However, our patient differed in several respects: though siltuximab has blocked IL-6 effectively devoting to the relief of fever, anemia and dyspnea, pulmonary hemodynamics (PVR 11.05 WU) and right heart size did not improve significantly. This dissociation suggests that in cases with advanced pulmonary vascular remodeling driven by chronic IL-6/JAK/STAT3 activation, controlling MCD activity alone may be insufficient to reverse established PH. Additionally, unlike most reported cases who received combination PH therapy at the early disease stage, our patient received macitentan as a late add-on targets the vasoconstrictive component via endothelin receptor antagonism, which may explain the further symptomatic relief independent from siltuximab treatment. Limited hemodynamic improvement may be related to the insufficient treatment duration of macitentan and siltuximab. These observations underscore the importance of early recognition and timely initiation of both immune-suppressive and PH-targeted therapies in this rare condition. Furthermore, PH that manifests within one year of MCD diagnosis appears to carry a worse prognosis.

Our case also provides several practical lessons. Firstly, unexplained dyspnea in MCD patients should prompt early echocardiography and right heart catheterization to avoid diagnostic delay. Secondly, timely combination therapy targeting both the underlying IL-6 driven inflammation (siltuximab) and pulmonary vasodilator (macitentan) may achieve better hemodynamic improvement than either alone. Finally, these immunocompromised patients are at high risk for infections, which can precipitate a fatal pulmonary hypertensive crisis; Therefore, close attention to infection prevention and timely initiation of treatment for suspected infections are critical.

The diagnostic process in this case still faced multiple challenges. First, the patient initially presented with non-specific systemic symptoms such as fever and lymphadenopathy accompanied by an EBV load of 190 copies/mL, which reasonably directed clinical attention toward EBV infection; however, persistent symptoms after anti-viral therapy suggested an underlying another etiology. Secondly, the early manifestations of PH were non-specific and easily masked by the systemic inflammatory state. It was not until the severe dyspnea developed and the examination of CT scan and echocardiogram the diagnosis of PH was definitively established. The diagnostic approach was therefore significantly delayed. Thirdly, the definite diagnosis of the etiology of PH relies on the histological confirmation of pulmonary vascular pathology. However, the biopsy of pulmonary artery carries substantial risks. Determining the possible etiology and contribution of MCD vs. EBV in driving PH was particularly challenging. The final etiology diagnosis was finally made based on the treatment response and multidisciplinary consultation.

Several limitations should be acknowledged. Firstly, as a single case report, the findings lack generalizability and definitive causal inference between MCD, EBV infection and PH. Secondly, while histological confirmation of pulmonary vascular pathology would ideally strengthen the mechanistic evidence, pulmonary artery biopsy carries substantial procedural risks and ethical concerns, which precluded the invasive approach in our patient. We therefore relied on the comprehensive clinical, imaging, and laboratory data to make definite diagnosis. Thirdly, for group 5 PH, establishing the precise contribution of EBV and MCD to pulmonary vascular remodelling remains challenging, and functional studies—such as animal models or *in vitro* mechanistic assays—would be required for definitive validation, which are beyond the scope of a single case report.

## Patient Perspective

**“**I have suffered from recurrent fever, sweat loss and fatigue for one year and I have been to hospital several times without receiving a definite diagnosis. I gradually felt dyspnea even though only 150 meters of walking distance. After the enlarged lymph node biopsy, multi-disciplinary consultation and target therapeutic approach, the symptom of dyspnea and recurrent fever were relieved.”

## Data Availability

The raw data supporting the conclusions of this article will be made available by the authors, without undue reservation.
