# Supplementary material for: Pulmonary hypertension in a patient with HHV-8–negative multicentric castleman disease and epstein-barr virus co-infection: a case report
Source: Front Med (Lausanne). 2026 Oct 14;13:1951176. doi: 10.3389/fmed.2026.1951176 (PMC13714884; doi:10.3389/fmed.2026.1951176)
Supplement: Supplementary file 1 [file Table1.docx]

**Supplementary table 1**

Patients with PAH complicated EBV and/ CD in previous case reports.

| **Age**  **gender** | **Mean PA**  **(mmHg)/**  **PVR(WU)** | **Complication** | **PAH medication** | **After treatment**  **mean PA**  **(mmHg)/**  **PVR(WU)** | **EBV/CD**  **treatment** | **Clinical**  **Outcome** | | | **DOI:** |
| --- | --- | --- | --- | --- | --- | --- | --- | --- | --- |
|  |  |  |  |  |  | **CD/EBV**  **resolved** | **PAH**  **resolved** | **Alive** |  |
| 9  male | estimated  60 mmHg | CAEBV | Sildenafil  Bosentan  Riociguat | estimated  40mmHg | PSL/CY  Thalidomide | **✔** | **✔** | **✔** | DOI:  10.3389/fped.2019.00219 |
| 44  male | 45/9.8 | CAEBV | / | 27/2.4 | CHOP  PBSCT | × | **✔** | × | DOI: 10.1536/ihj.19-419 |
| 11  male | 43/9.6 | CAEBV | Sildenafil  Bosentan  beraprost | NA | PSL/CsA  PBSCT | Not sure | × | × | DOI: 10.1111/ped.12578 |
| 41  male | 64/13.3 | CAEBV | Sildenafil  Epoprostenol | 61 | PSL/CsA  VP-16 | Not sure | × | × | DOI: 10.2169/internalmedicine.50.4143 |
| 36  female | 65/19.1 | CAEBV | Epoprostenol | 15/1.9 | fludarabine  melphalan  irradiation  CBT | **✔** | **✔** | **✔** | DOI:  10.1038/  bmt.2014.129 |
| 7/female | estimated  35 mmHg | CAEBV | Bosentan | normal | ESCAP | **✔** | **✔** | **✔** | DOI:  [10.3760/cma.j.cn112140-20210718-00594](https://doi.org/10.3760/cma.j.cn112140-20210718-00594) |
| 23/female | 40/4.6 | CAEBV | Tadalafil | NA | CHOP  ESCAP  BMT | **✔** | **✔** | **✔** | DOI:  10.1016/j.jiac.2022.10.013 |
| 41/female | 55/16.4 | CAEBV | Tadalafil  Ambrisentan | 32/3.7 | CHOP  AraC  BMT | × | **✔** | × |  |
| 69/female | 85/18.5 | CAEBV | Tadalafil  Macitentan | 36/4.5 | GDP | × | **✔** | × |  |
| 30/female | 31/5.8 | CAEBV | Tadalafil  Macitentan  Selexipag | NA | CHOP  BMT/CBT | Not sure | **✔** | × |  |
| 34/female | 58/13.9 | CD+HHV-8 | Prostacyclin | 63/13.1 | CY  Decadron  Rituxan | **✔** | × | × | DOI:  10.1183/09031936.03.00006903 |
| 44/male | 48/5.3 | CD | Prostacyclin  Bosentan | 28/2.5 | Rituxan | ✔ | ✔ | ✔ |  |
| 31/female | 64/15 | CD+HIV  HHV-8 | Epoprostenol | 22/2.4 | Zidovudine  Lamivudine  Saquinavir  Ritonavir  CY | ✔ | ✔ | ✔ | DOI: 10.1183/09031936.05.00133904 |
| 31/female | 49/10.8 | CD | Beraprost sodium  Bosentan  Sildenafil | / | PSL  Tocilizumab | ✔ | × | ✔ | DOI:  10.1007/s00380-009-1215-5 |
| 38/male | 32/NA | CD | / | / | Tocilizumab  plasmapheresis | ✔ | ✔ | ✔ | DOI 10.1007/s12185-009-0346-x |

**Supplementary table legends:**

PVR, pulmonary vascular resistance; PAH, pulmonary artery hypertension; CAEBV, chronic active Epstein-Barr virus infection; NA, not available; CHOP, cyclophosphamide, doxorubicin, vincristine and prednisolone; PSL, prednisolone; CsA, cyclosporine A; VP-16, etoposide; ESCAP, etoposide, cytarabine, L-Asparaginase, methylprednisolone (mPSL); CY, cyclophosphamide; AraC, cytarabine; PBSCT, peripheral blood stem cell transplantation; BMT, bone marrow transplantation; CBT, cord blood transplantation; GDP, gemcitabine, dexamethasone, cisplatin; NA, no data; CD, Castleman disease; HIV, Human Immunodeficiency virus; HHV-8, Human Herpesvirus-8
